# Cold Feet

**Published:** 1861-01

**Authors:** 


**COLD FEET.**—It is impossible to have vigorous health if the feet are habitually cold; no amount of external covering can keep them warm. Wearing pepper and other irritants in the stockings, is generally inefficient, is always hurtful in its tendencies, and never accomplishes a permanent radical good. One of the most uniformly efficient means of keeping the feet warm is to wash them in water at least as cold as the atmosphere of the room, night and morning; let it be done within a minute in very cold weather, then wipe and rub them rapidly and thoroughly with a very coarse towel, dress, and when practicable, take a walk, or dry them by the fire, rubbing them well with the hands.

In addition, let half an inch of curled hair be basted to a piece of cloth and slipped in the stocking, the hair touching the soles of the feet to titillate the skin, and thus aid in drawing the blood thither to warm them. The hair conducts the moisture from the feet to the woolen cloth, and thus keeps them dry. These hair-soles should be placed before the fire at night, so as to be thoroughly dried by the morning. Cork-soles absorb moisture from the shoe and the feet also, and require several days to be thoroughly dried. India-rubbers confine the dampness about the feet, hence they should be promptly removed as soon as the wearer ceases walking, nor should they be used except in muddy, slushy weather.

